# Women's empowerment and fertility preferences of married women: analysis of demographic and health survey’2016 in Timor-Leste

**DOI:** 10.3934/publichealth.2022017

**Published:** 2022-01-12

**Authors:** Nandeeta Samad, Pranta Das, Segufta Dilshad, Hasan Al Banna, Golam Rabbani, Temitayo Eniola Sodunke, Timothy Craig Hardcastle, Ahsanul Haq, Khandaker Anika Afroz, Rahnuma Ahmad, Mainul Haque

**Affiliations:** 1 Department of Public Health, North South University, Dhaka, Bangladesh; 2 Department of Statistics, University of Dhaka, Dhaka, Bangladesh; 3 Institute of Social Welfare and Research, University of Dhaka, Dhaka, Bangladesh; 4 Health Systems and Population Studies Division, International Centre for Diarrhoeal Disease Research, Bangladesh (icddr,b), Dhaka, Bangladesh; 5 Department of Anatomy, University of Ilorin, Nigeria; 6 Department of Surgery, University of KwaZulu-Natal, South Africa; 7 Gonoshasthaya-RNA Molecular Diagnostic & Research Center, Dhanmondi, Dhaka-1205, Bangladesh; 8 Deputy Manager (Former), Monitoring, Learning, and Evaluation, CEP, BRAC, Bangladesh; 9 Department of Physiology, Medical College for Women and Hospital, Dhaka, Bangladesh; 10 Unit of Pharmacology, Faculty of Medicine and Defence Health, Universiti Pertahanan Nasional Malaysia (National Defence University of Malaysia), Kem Perdana Sugai Besi, 57000 Kuala Lumpur, Malaysia

**Keywords:** women's empowerment, enablement, fertility preference, predilection, fertility rate, frequency, Timor-Leste

## Abstract

A recently independent state, Timor-Leste, is progressing towards socioeconomic development, prioritizing women empowerment while its increased fertility rate (4.1) could hinder the growth due to an uncontrolled population. Currently, limited evidence shows that indicators of women's empowerment are associated with fertility preferences and rates. The objective of this study was to assess the association between women empowerment and fertility preferences of married women aged 15 to 49 years in Timor-Leste using nationally representative survey data. The study was conducted using the data of the latest Timor-Leste Demographic and Health Survey 2016. The study included 4040 rural residents and 1810 urban residents of Timor-Leste. Multinomial logistic regression has been performed to assess the strength of association between the exposures indicating women's empowerment and outcome (fertility preference). After adjusting the selected covariates, the findings showed that exposures that indicate women empowerment in DHS, namely, the employment status of women, house and land ownership, ownership of the mobile phone, and independent bank account status, contraceptive use, and the attitude of women towards negotiating sexual relations are significantly associated with fertility preferences. The study shows higher the level of education, the less likely were the women to want more children, and unemployed women were with a higher number of children. Our study also found that the attitude of violence of spouses significantly influenced women's reproductive choice. However, employment had no significant correlation with decision-making opportunities and contraceptive selection due to a lack of substantial data. Also, no meaningful data was available regarding decision-making and fertility preferences. Our findings suggest that women's empowerment governs decision-making in fertility preferences, causing a decline in the fertility rate.

## 1. Introduction

Timor-Leste is a Southeast Asian nation-state principally publicly sponsored healthcare and provides without any copayment at the point of use [1]. Thereby, removing the payment system from public healthcare improves immensely utilization and access to state-owned primary-care and medical care with overall medical management among Timor-Leste citizens [1]–[3]. As described by World Health Organization (WHO), the reason was that out-of-pocket health expenses reduce to below 10% among healthcare seekers of the country [2]. The removal of copayment and improving public healthcare access and utilization are similarly observed in many other low- and middle-income countries (LMICs) [4]–[6]. However, like many other countries, Timor-Leste fails to eliminate health inequalities regarding access to the public healthcare system [7]–[10].

Nevertheless, the government of the República Democrática de Timor-Leste (Democratic Republic of Timor-Leste) developed a strategic plan for the country's overall development that health, education, and many other vital issues of the country [11]. Furthermore, multiple studies reported that the country has improved in different human development indexes, including the health sector with wholesale medical and primary health care in the post-conflict era [12]–[16].

However, the United Nations Population Fund (UNFPA), Plan International, Government of Timor-Leste jointly reported that In Timor-Leste, almost one in five teenage girls start their marital life before 18, and 24% of them get pregnant and deliver a child before the age of 20 [17]. Similarly, another reported that teenage girls' sexual activity and pregnancy are frequently observed in East Asia and the Pacific, including Timor-Leste, in the milieu of low contraceptive prevalence [18]. The situation started improving [19], but still, less than 10% of married adolescents without children practice modern methods of contraception (mDFPS) in many LMICs that Timor-Leste [20]. It has been reported that teenage girls of Timor-Leste, especially of marginalized communities of rural areas added with unschooled, have a higher-level burden of child (15–19 years) pregnancy than those who were financially solvent, higher educational level, and living in metropolis [1],[21]. Additionally, In Timor-Leste intimate partner violence is more than 20% [22]. Another study revealed that only 10.8% of Timorese women suffer no physical violence from an intimate partner. Rest Timorese women suffer from low respect/regard and no abuse (32.9%), severe psychological abuse (30.6%), physical abuse only (6.2%), and Physical abuse + severe psychological abuse (19.5%) [23]. Barlake—an indigenous dominant socio-cultural practice of Timor-Leste. Multiple international agencies have reported that such indigenous traditional activities are the root cause of childhood marriage, teenage pregnancy, physical violence, gender inequity, and many more issue of the country [18],[24]–[27]. Nevertheless, multiple anthropological studies revealed that these statements have often been deficient in an in-depth interpretation of community perspective and conventional socio-cultural system [28],[29].

The term women's empowerment refers to women's decision-making authority, which creates an avenue for them to exert their right to use contraceptives and other fertility preferences [30]–[32]. Worldwide the fertility rate decreased from 3.2 live births per woman in 1990 to 2.5 in 2019. Additionally, it has been reported that around half of the global population has lifetime fertility below 2.1 live births per woman, which tends to control population density throughout the world with low mortality to have a growth rate of zero in the future and ensure standard socioeconomic status [33]. However, global public health opinion leaders have observed a gap in rates and preferences of fertility between high-income countries (HICs) and LMICs [34]–[37]. In developing countries, evidence demonstrates that fertility rates are now reducing with the availability of modern contraceptive methods [38]–[42]. Therefore, the decision to adopt such practices indicates empowerment to some extent. It has been noticed that in the LMICs, women's empowerment promotes lower fertility preferences, high education, and rapid economic growth [43]–[46].

The prioritized preferences for women's empowerment associated with fertility include education, skills development, independent decision-making ability, and control over household resources [43],[47],[48]. Multiple studies revealed from a more positive angle that improved level of employment and access to and control over resources have significantly reduced the ideal number of children given birth [43],[49],[50]. In general, evidence shows that higher educational accomplishments significantly correlate with lower fertility rates [18],[51]–[53]. It has been revealed that working women with an optimal level of education tend to prefer a limited number of children in the African countries possessing high fertility rates [43],[50],[54],[55].

Furthermore, in Southern Asia, a similar trend for women's empowerment has resulted in progressive paths to lower fertility, longer birth intervals, and lower rates of unintended pregnancy [47],[56]. Fertility behavior, education of women, employability, active decision-making, advocacy through media and spousal communication in India [57] has been significantly impacted by women's empowerment, which also aligns with the scenario in Asian countries, namely, Cambodia [58], Indonesia [59], Philippines [60], Nepal and China [61], Vietnam [62], Afghanistan, Bangladesh, India, Nepal, and Pakistan [63] and Timor-Leste [64]–[67]. The World Bank report shows that South Asia's female reading ability rate has improved from 45.5% to 57% between 2000–2010 [52]. A declining movement of the total fertility rate was detected from 6 in 1960 to 2.6 in 2014 [52],[68]. Surprisingly, the sex of the first child is a critical issue of the families of South Asia, and a boy child is most expected [69]–[72]. Those women who deliver boys enjoy more independence or self-governance than others of the opposite sex, leading to having more children one after another if their sex is female with the hope of having a boy [56],[73]–[75].

Women's proprietorship of household and agricultural land plays a significant role in decision-making authority, linked to a lower fertility rate [43],[50],[76],[77]. Moreover, women with greater autonomy in decision-making can influence and negotiate safer sex practices, especially those in nuclear households [78]–[81]. This right of being ascertained to have greater control of power also helps resolve disagreements over contraception that might be a potential threat from their partners as fertility preferences are significantly impacted by violence [82]–[86]. It is noteworthy that women who stand up against any form of abuse have a higher likelihood of using modern contraceptives [87],[88]. Thereby, fertility preference is shifted from having more children to having a lesser number of children. Another significant determinant that plays a vital role in fertility preferences is the mother's age [89].

This study mainly focuses on Timor-Leste, a recently independent South East Asian state recognized by the UN in 2002, through the approved constitution of the Democratic Republic of East Timor [90]. Timor-Leste is progressing with socioeconomic development as a recent sovereign country, while evidently, women's education and overall domestic upgrading improve women's empowerment [66]. However, in some parts of Timor-Leste, the number of antenatal clinics (ANC) visits exponentially rose with an increased labor force participation and the rise in advocacy against violence/assault posed by men (physical abuse), levels of women's education, and decision-making influences [67],[91]. As an example, wealthier and highly educated married women were more likely to refuse sexual intercourse and even tell their partners to use a condom; thus, gaining control over their fertility preference [80],[92],[93]. The rate of sexual violence is significantly high in Timor-Leste, especially among women living in rural areas [94]. It is commonly believed that these encounters often result in unplanned pregnancies, negatively impacting fertility preference [95],[96].

Moreover, a Timorese woman can rarely exercise her right to access contraception freely and independently [97],[98]. Also, fertility preferences are sometimes influenced by religious leaders. Even the choice of contraception is mainly constrained by family, culture, tradition, and educational influences [97]. Interestingly, interactions with the wealthy, such as those who own mobile phones, have better access to maternal health care services. In contrast, underutilization of maternal health care facilities is associated with poverty and lack of education [67],[91],[99],[100].

Women's empowerment and its influence on contraception, fertility, family planning, and other maternal care-related aspects have been explored in LMICs in Asia and Africa to a certain degree [101],[102]. The ongoing women's empowerment campaigns in Timor-Leste probably influenced fertility preference [97]. Although, the current fertility rate decreased to 6.18 in 2002 from 3.9 in 2020 in Timor-Leste, which is an improvement for the country after independence [103]. Furthermore, land, house, mobile phone and bank account ownership by women and negotiation of sexual relations had not been widely explored while assessing women's empowerment and its impact on reproduction [104],[105]; the decision-making process, control over the household, contraceptive use, skill development, education, and employment had been the key contributors to women's empowerment in most prior studies [48],[83],[97],[106]–[110]. Therefore, this study aims to assess the association of women's empowerment considering the overlooked exposures and fertility preferences in Timor-Leste by analyzing the Demographic and Health Survey Data from 2016 so that the findings may assist policymakers in adjusting and modifying policies for a better outcome in terms of population control and overall development.

## 2. Materials and methods

### 2.1. Data source and study settings

The study analyzed the 2016 Timor-Leste Demographic and Health Survey (TLDHS) data, collected between September 16–22, 2016.

### 2.2. Study design and study populations

The TLDHS 2016, implemented by the General Directorate of Statistics (GDS) of the Ministry of Planning and Finance in collaboration with the Ministry of Health (MOH), was used for this study. The TLDHS 2016 used the sampling frame, which was used for the 2015 Timor-Leste Population and Housing Census (2015 TLPHC) provided by the Timor-Leste GDS. A representative probability sample of approximate size 12,000 was selected (TLDHS, 2016). In the first stage, 455 enumeration areas (EAs) were selected with probability proportional to EA size from the 2015 TLPHC consisting of 129 EAs in urban areas and 326 EAs in rural areas. In the second stage, within each of the 455 EAs, 26 households were randomly selected. The anticipated rates of non-response at the household and individual levels were considered at the time of sample design and sample size calculations. To prevent bias, no replacement of pre-selected households was allowed. All the selected households were eligible for interviews. All women aged 15–49 who were either usual residents or visitors of the selected households were interviewed.

### 2.3. Variables and their measurement

The women's employment status was measured from two indicators: currently working or not and had a job but presently absent. The women saying “Yes” to any of the indicators was considered as “Employed”; otherwise, they were marked as “Unemployed”. The status of owning land or house was measured using two indicators: owns a land alone or jointly, or owns a house alone or together. The variable owning area or house was coded as “No” if the women-owned neither land nor house, otherwise coded as “Yes” The decision making of the women's empowerment were appraised utilizing three indicators: i. who is decision maker regarding the medical care of wife, ii. making large household purchases, and iii. wife's socialization or visiting parenteral house and family or relatives. According to the DHS guideline: i. Active participation means women participate in decision-making alone or jointly with her husband on all three indicators; ii. otherwise, she was considered not participating in decision-making. Women who had used the internet in the last 12 months were considered internet users. Women who had ever used something to delay or avoid pregnancy were deemed to be contraceptive users. The variable marital control exercised by the husband was coded as “Yes” if the women said yes to any of the indicators, otherwise “No”. Marital control is defined as the “percentage of ever-married women age 15–49 whose current husband/partner for currently married women or most recent husband/partner for divorced, separated, or widowed women ever demonstrated each of the following controlling behaviors”: envious or annoyed if she dialogs to other men; recurrently indicts her of being disloyal; does not allow her to see her female boon companion; ongoing effort to restrict her to interact with her parents and siblings; every moment is chasing her about where about; does not believe her in financial issue [111]. Women were asked whether she is justified to ask the husband to use condoms if he has STI, refuse to have sex if she is tired, and refuse to have sex if the husband has other women. If the women said “Yes” to any of those situations, the variable “negotiating sexual relations” was coded as “Justified” for that woman otherwise “Unjustified”. The variable regarding owning a mobile phone and an independent account at a bank or other financial institution had categories “Yes” and “No”. In this study, employment status, owning land or house, decision making, attitude toward wife beating, internet use, contraceptive use, marital control exercised by husband, negotiating sexual relations, owning a mobile, and owning account are the variables considered indicators of women's empowerment.

Only three categories for potential fertility preferences were offered for this study: Have another, Undecided, and No more. Age was categorized as 15–24, 25–34, 35–44, and more than 45. The variable number of living children was classified as 0–3, and more than 3. The husband's occupational status was categorized as unemployed, agricultural, and non-agricultural. Furthermore, variables regarding women's essential characteristics, such as their residence, level of education, husband's education level, and household socioeconomic status, were also considered.

### 2.4. Sample size

To get statistics that represent Timor-Leste, the distribution of the women in the sample needs to be weighted (or mathematically adjusted) to reflect the true distribution in Timor-Leste. Women from a small municipality, such as Aileu, should contribute a smaller amount to the national estimates based on the total sample. Women from a large metropolis, such as Dili, should contribute much more. Therefore, DHS statisticians mathematically calculate a “weight”, which is used to adjust the number of women from each municipality so that each municipality's contribution to the total is proportional to the actual population of the municipality. The weighted values can be smaller or larger than the unweighted values at the municipality level. The total national sample size of 12,607 women has not changed after weighting. Still, the distribution of women across municipalities has been changed to reflect their actual contribution to the total population size. We used 5850 participants' data for this study after dropping the missing observation from the central database [111],[112].

### 2.5. Statistical analysis

The DHS data were first filtered using variable marital status, usual resident or not, currently pregnant or not, fertility preferences, husband's education level, and occupation group. Only the data of married women who are not pregnant or unsure about their pregnancy status as well as her fertility preferences are have another or undecided or no more, and whose husband's education level, as well as husband's occupation group, was correctly identified were considered for the analysis. After filtering the raw data set, data from 5850 women were obtained, but the weighted sample size was 5878. Most of the analysis was based on data from the 5878 women, except for some variables where the analysis was performed after removing missing responses and responses like “do not know”. All the analysis was done after incorporating the sample weight and the survey design of DHS. A robust significant association (p < 0.001) was noted in sensitivity analysis between the number of children and fertility preference. Thus, the OR was estimated after stratification of the number of children.

Some covariates were selected for this study, which could be associated with fertility preferences. These covariates include age group (15–24, 25–29, 30–34, 35–39, 40–49), place of residence (Urban, Rural), level of education (No education, Primary, Secondary, Higher), husband's education level (No education, Primary, Secondary, Higher), husband's occupational group (Unemployed, Agricultural worker, Non-agricultural worker) and household socioeconomic status (Poorest, Poorer, Middle, Richer, Richest). A significant difference was noted between families with more minor than and more than 3 living children. Thus, after including all the predictor variables in the same model, a stratified analysis was performed.

The univariate chi-square test was performed between fertility preferences and all the covariates selected. The multivariate logistic regression model was fitted to know the effect of each women's empowerment indicator in the presence of all the covariates found to be associated with fertility preferences. Hosmer–Lemeshow test was used to determine whether the fitted model adequately described the observed outcomes. Those covariates were kept in the model regardless of their p-value. All the p-values <0.05 were considered significant. The analyses were performed using software STATA version 15 (StataCorp, LP, College Station, Texas, USA) and SPSS 22 (IBM SPSS Statistics for Windows, Version 22.0. Armonk, NY: IBM Corp.).

## 3. Results

### 3.1. Sample socio-demographic characteristics

The general socio-demographic characteristics of the respondents are depicted in Table 1. In the sample, most women belong to 25–34. About 69.06% and 30.94% of women are rural and urban areas. In terms of the DHS, participant education level was mainly in the secondary education group (43%), as was the majority (40.1%) education level of their husband's. Research respondents employed and unemployed were 57.3% and 42.7%, respectively. Again, this study respondent's husbands worked largely (49.7%) in the non-agricultural sector. Most women's household socioeconomic status was almost equally divided across the various income categories. Contraceptive use was found at 38.8%. Most women had one or more children, at 96.3% combined.

### 3.2. Distribution of living children based on socio-demographic information

The current study revealed that most women wanted no more were highest among women with more than 3 children. Similarly, those wishing to have other children were highest among women with 1–3 children. Women with more than three children participating in the overall decision-making process were 45.9% and 50.6% among women with 1–3 and more than 3, respectively. Contraceptive use was more observed among women having more than 3 children (Supplementary Table 1). This study also revealed that 70.1% and 68% of women want to have another child and no age range 25–34, and 45 and above, respectively (Supplementary Table 2).

**Table 1.** General socio-demographic characteristics of the study respondents.

Variables	Overall (n = 5850)	Urban (n = 1810)	Rural (n = 4040)
Age range in years (Mother information)			
			
15–24	700 (12.0%)	179 (9.89%)	521 (12.9%)
25–34	2576 (44.0%)	873 (48.2%)	1703 (42.2%)
35–44	1953 (33.4%)	596 (32.9%)	1357 (33.6%)
45 and above	621 (10.6%)	162 (8.95%)	459 (11.4%)
Education			
No education	1748 (29.9%)	247 (13.7%)	1501 (37.2%)
Primary	1148 (19.6%)	228 (12.6%)	920 (22.8%)
Secondary	2513 (43.0%)	1003 (55.4%)	1510 (37.4%)
Higher	441 (7.54%)	332 (18.3%)	109 (2.70%)
Employment status			
Unemployed	3351 (57.3%)	971 (53.7%)	2380 (58.9%)
Employed	2499 (42.7%)	839 (46.4%)	1660 (41.1%)
Distribution of living children			
No children	214 (3.66%)	76 (4.20%)	138 (3.42%)
1–3	2704 (46.2%)	906 (50.1%)	1798 (44.5%)
More than 3	2932 (50.1%)	828 (45.8%)	2104 (52.1%)
Husband information			
Education level			
No education	1595 (27.3%)	235 (13.0%)	1360 (33.7%)
Primary	1223 (20.9%)	247 (13.7%)	976 (24.2%)
Secondary	2348 (40.1%)	888 (49.1%)	1460 (36.1%)
Higher	684 (11.7%)	440 (24.3%)	244 (6.04%)
Employment status			
Unemployed	1296 (22.2%)	293 (16.2%)	1003 (24.8%)
Agricultural work	1649 (28.2%)	166 (9.17%)	1483 (36.7%)
Non-agricultural work	2905 (49.7%)	1351 (74.6%)	1554 (38.5%)
Household socioeconomic status			
Poorest	1057 (18.1%)	53 (2.93%)	1004 (24.9%)
Poor	1159 (19.8%)	96 (5.30%)	1063 (26.3%)
Middle	1217 (20.8%)	218 (12.0%)	999 (24.7%)
Richer	1311 (22.4%)	622 (34.4%)	689 (17.1%)
Richest	1106 (18.9%)	821 (45.4%)	285 (7/05%)
Contraceptive use	2270 (38.8%)	727 (40.2%)	1543 (38.2%)
Domestic violence (Beating women)			
Justified	4095 (77.6%)	1240 (75.5%)	2855 (78.6%)
Not justified	1180 (22.4%)	403 (24.5%)	777 (21.4%)

*Note: Data was presented as a number with percent in the parenthesis.

**Table 2.** Frequency of living children based on socio-demographic information on empowerment factors.

Variables	No children	1–3 children	>3 children
Fertility preference			
No more	6 (0.33%)	390 (21.2%)	1444 (78.5%)
Undecided	74 (3.93%)	803 (42.6%)	1008 (53.5%)
Expected to conceive	134 (6.31%)	1511 (71.1%)	480 (22.6%)
Have another	-	1511 (71.1%)	480 (22.6%)
Decision			
Do not participate	31 (4.51%)	336 (48.9%)	320 (46.6%)
Participate	183 (3.54%)	2368 (45.9%)	2612 (50.6%)
Contraceptive use			
No	206 (5.75%)	1633 (45.6%)	1741 (48.6%)
Yes	8 (0.35%)	1071 (47.2%)	1191 (52.5%)

*Note: Data was presented as a number with percent in the parenthesis.

**Table 3.** Stratified fertility preference based on age range of the study participants.

Variables	No more	Undecided	Have another
Age range in years (Mother information)			
			
25–34	21 (3.00%)	188 (26.9%)	491 (70.1%)
15–24	412 (16.0%)	860 (33.4%)	1304 (50.6%)
35–44	985 (50.4%)	673 (34.5%)	295 (15.1%)
45 and above	422 (68.0%)	164 (24.6%)	35 (5.64%)

*Note: Data was presented as the number with percent in the parenthesis.

### 3.3. The comparison was made on the fertility preference

**Figure 1.**
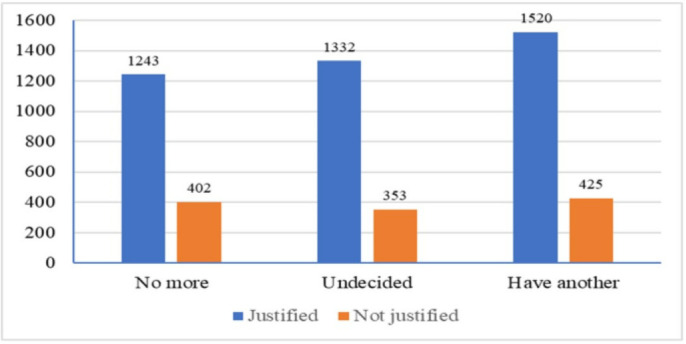
Illustrates women facing gender violence and the expectation of another child.

The spouse who had the attitude of violence against women showed a more significant influence on women's reproductive choice. Infertility preference the spouse who thinks justified to beat their wife had ~80.0% influence on the reproductive choice (Figure 1).

**Figure 2.**
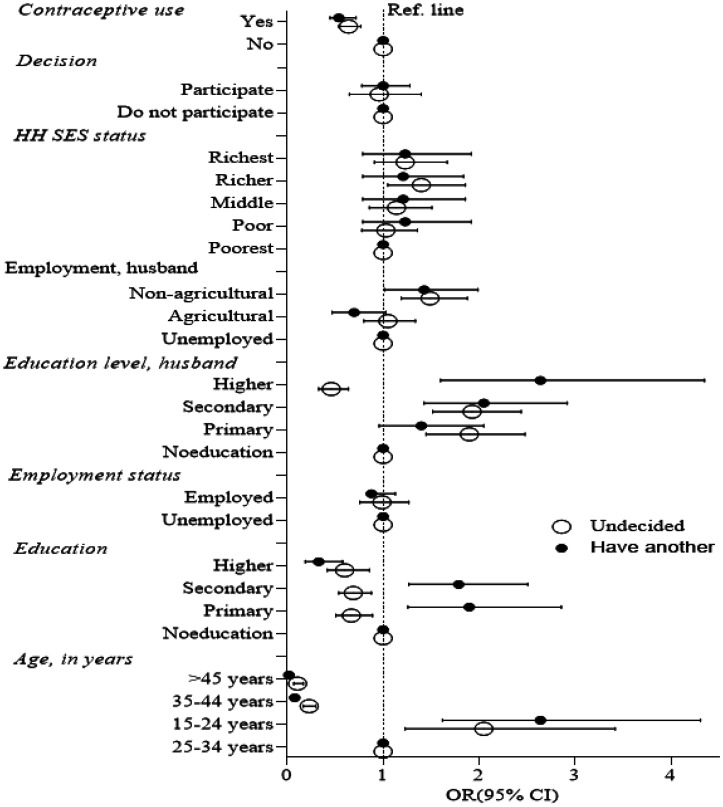
Odds of fertility preference among the participants who had less than 3 living children.

**Figure 3.**
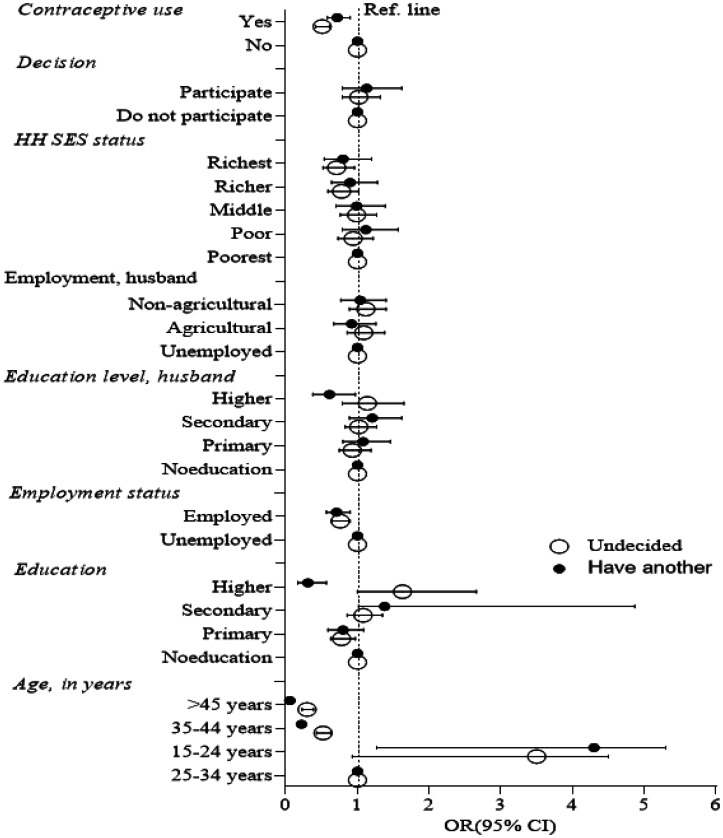
Odds of fertility preference among the participants with more than 3 living children.

The comparison was made on the fertility preference of no more child than undecided and having another child. The line indicates the reference line of an odds ratio. Data were shown as OR with a 95% confidence interval. The multivariate logistic regression model was used to estimate the OR and p-values. The regression model was performed after including all the variables in the model (Figure 2 and Supplementary Table 1). The comparison was made on the fertility preference of no more child than undecided and having another child. The more education women with fewer than 3 children have, the less likely they want another child. The higher the education, the more husbands want children among those with fewer (Figure 3 and Supplementary Table 2). While women are employed, they are significantly more undecided and less likely to want another. Interestingly, in contrast to those fewer than three, women with higher education husbands are also substantially less likely to want another. Still, their employment status is not significant (Figure 3).

## 4. Discussion

This study provides a new understanding of the association between women's empowerment in Timor-Leste and their fertility preferences utilizing a nationally representative survey, TLDHS. The findings were analyzed with a multinomial logistic regression model with adjusted covariates. After adjusting covariates, it is statistically evident that several variables concerning women's empowerment are significantly associated with the fertility preferences of the studied population.

This study revealed that most research respondents were from rural Timor-Leste, and most of them were from the age group of 25–34. A considerable number came from the age group of 15–24. Based on secondary data [2016 TLDHS data], this study, where 15–18 years old married females are considered women instead of children, shows the problematic child marriage situation in Timor-Leste. The 1990 Convention on the Rights of Child (CRC), a legally-binding global arrangement endorsed by almost all counties [113]. This promulgation protects children under the age of eighteen years [113]. Child marriage, defined by the United Nations and CRC as marriage before the age of 18, is well thought-out as a desecration of human rights with negative significances for child health globally [114],[115]. Multiple studies around the globe observed that child marriage exists [115]–[118], which promotes health consequences [116]–[121]. One Indian study reported that child marriage significantly upsurges the risk of childhood anemia in adjusted analyses (AOR = 1.08, 95% CI = 1.03–1.13) [116]. Another Asian study similarly reported that malnourished children correlate with mothers' early age marriage, enlightening level, and nutritious standing [117].

United Nations Children's Fund (UNICEF) reported situation is too grave. It was estimated in 2018, 650 million girls and women living on our planet married before their 18th birthday [122]. The practice of child marriage is documented as a significant barricade of mother and child health and overall global health [93],[114],[123],[124]. Additionally, child marriage or marriage without both spouses' free and full consent is a human rights violation [125]. The studies reported that gender inequality, poverty, social injustice, and insecurity lead to educational restrain, reduced economic opportunities, increased risk of domestic violence, facing early, frequent, and very high-risk pregnancies in Timor-Leste [95],[96],[126],[127]. Child marriage restrains their education, reduces their economic opportunities, increases the risk of domestic violence, and puts them at risk for early, frequent, and very high-risk pregnancies [127],[128]. Consequently, girl marriage significantly impacts women's empowerment below 18 [129]–[131].

This study found that women's reproductive choice was influenced by the attitude of violence of their spouses. The spouses with an attitude of violence had a more significant influence on women's reproductive choices. The current study findings were in the same line of a similar study conducted in Sub Saharan Africa also found that women's decision-making was negatively influenced by intimate partner violence [132]. One study reported that bodily brutality among less-empowered, nullipara women was more than that of more-empowered women with only male children. Less-empowered, unschooled women were more vulnerable to increased corporeal ferocity than more-empowered, primary-educated females [133]. This present study was performed on the parameters mentioned above, which only reaffirms the findings of foundations like the Asia Foundation [134],[135] which reported that Timor-Leste's national economy, along with the economics of marriage, is also exceedingly gendered biased and full of inequity. Although, women in Timor-Leste repeatedly contribute to various income-generating accomplishments and weaving.

Nevertheless, women labor-force were much less paid typically than their male counterparts. Women's earnings are principally spent for family wellbeing and children. Therefore, women's empowerment and decision-making on women's fertility choices in Timor-Leste were based on these issues [78].

Education remains the most powerful tool of empowerment and improves the status of any individual irrespective of sex, especially among women [136]–[138]. The current study shows that the higher the education, the less likely women want another child. Women with a primary and secondary level of schooling want more children, especially those with less than three living children. This study also shows that having children is higher in unemployed women than in employed ones. This finding is similar to recent studies conducted in India, other studies using national data in Southeast Asia, particularly in Cambodia and modern high-income countries (HICs) [66],[139]–[142]. Employed women can significantly participate in the decision-making process, an essential indicator of women's empowerment while favoring decision-making regarding fertility issues [83],[88]. Employment creates self-reliance, enabling women to participate in decision-making [143],[144]. They are found to be less likely to have more children than unemployed women having no participation in decision-making [43],[50],[66],[145]–[147]. However, this study shows no significant difference in employment and decision-making opportunities of Timorese women. Even if it is seen that employed women are less likely to have more children, the number of living children does not have any significant impact on the ability to participate in the decision-making process, statistically.

Meanwhile, multiple studies observed that women enjoy more autonomy in activities like family planning as economic associations and interactions that involve nuclear family settings than a joint family structure [48],[81]. Despite showing a clear correlation between employment status and child preference [81],[148],[149], the current study could not show any significant data to prove a correlation between employment status and decision making or selection of contraceptives.

The current study tried to relate contraception and fertility preferences with women's decision-making ability, leading to empowerment. The database showed no significant data on decision-making and fertility preferences. Also, there is no linear correlation between women's empowerment and fertility preferences; it is influenced by circumstantial factors such as family, cultural, traditional, and educational factors, despite the wishes of Timorese women to access their right to contraception [97]. According to various studies conducted in Timor-Leste and other LMICs, it was stated that contraceptive use depends on knowledge of methods, desire to use them, fear of health impacts, religious beliefs, support from partners, suppliers, and resources [97],[150],[151].

The current study shows that 38.8% of respondents use contraceptives. Additionally, 31.4% want no more children, and 32.2% are undecided on whether they want another child. There is no available data in the given database about the type of contraceptive they use or who chooses the contraception method. Contraception can be hormonal, permanent surgical, barrier methods [152],[153], natural methods such as tracking the menstrual cycle, abstinence, understanding between couples, and indigenous practices [154]. Wallace et al. (2018) [97] said that the traditional Timorese marriage ritual barlake, which involves gifts or money from a groom's family to a bride's family when they marry, have a massive impact on child preferences; some of the residences think the amount they give to the bride's family during marriage gives them right to demand as many children they want. That study [97] also found that women need their husbands' permission to use contraceptives; otherwise, it may increase domestic violence. Men's prohibition of contraception has been associated with perceptions that its use undermines a husband's authority within a family [155]. Power and status in Timor-Leste are linked with notions of masculinity [156]. Religious belief is another influence on the Timorese fertility rate. As 70% of them are Roman Catholic Christian, they believe they have to live as a couple, conceive immediately after marriage, and bring children to the world [126].

A study conducted in Ethiopia found that a higher rate of contraceptive use and control of fertility is notable for well-educated women, against all violence-related atrocities, able to access media platforms or those from a wealthy background [157]. To further support this, Indonesia, a Southeast Asian country, has shown a higher contraceptive prevalence, thus lowering the fertility rate by 2.28 [158], which helps in Timor-Leste [159],[160]. However, a study in India discovered a link between sexual violence and its likelihood of causing a reduction in contraceptive use [86].

### 4.1. Limitations

The study only considered married women as respondents, while the association between women's empowerment and fertility preferences could also be demonstrated among women irrespective of marital status. Moreover, the socio-demographic factors selected as covariates in this study might not be adequate, as there could be several other possible covariates that may interfere with the association. Another limitation of the study is that only the association between indicators of women's empowerment and fertility preferences has been assessed, but causal inference cannot be drawn. This was a cross-sectional study with its inherent shortcomings. The study result cannot be utilized to investigate comportment over time.

Moreover, it does not help pin down cause and effect. The findings are a motionless picture, thereby unable to ensure to be representative. The Neyman bias occurs when we estimate the prevalence or incidence. This study tried to reduce any bias by no replacement of pre-selected households was allowed. All the selected families were eligible for interviews.

## 5. Conclusions

The evidence that women's empowerment is associated with fertility preferences in Timor-Leste, implying that an empowered woman tends to have fewer children than an unempowered woman. The government of Timor-Leste has executed a program to ensure gender impartiality and women's rights as per United Nations policy. Moreover, the government has taken different initiatives, including creating the new constitution to safeguard women's human rights better and forming a working group to support women. The findings of this study are believed to play a crucial role in promoting the program and stimulating the policymakers and concerned personnel of the government to establish women's empowerment not only to encourage development but also to control population growth. Thus, policy recommendations based on our study findings are to a) ensure equal employment opportunities in both public and private sectors, b) ensure access to finance and technologies, c) ensure land tenure rights, and d) ensure rights to sexual and reproductive health. The study asserts that adequately implementing the above policies with the government's current program will be a new perspective to achieve desired family planning and reproductive health outcomes, including transformational and sustainable change in gender mainstreaming in contemporary Timor-Leste. This study, along with future research, will help ensure incorporating freedom in fertility preferences and controlling the increased fertility rate in Timor-Lester and other high fertility countries in a broader perspective.
