# Supplementary material for: Women's empowerment and fertility preferences of married women: analysis of demographic and health survey’2016 in Timor-Leste
Source: AIMS Public Health. 2022 Jan 12;9(2):237–61. doi: 10.3934/publichealth.2022017 (PMC9114782; doi:10.3934/publichealth.2022017)
Supplement: Supplementary file 1 [file publichealth-09-02-017-s001.pdf]

Public Health

AIMS Public Health, 9(2): 237–261.
DOI: 10.3934/publichealth.2022017
Received: 28 October 2021
Revised: 26 December 2021
Accepted: 04 January 2022
Published: 12 January 2022

http://www.aimspress.com/journal/aimsph

Research article

Women's empowerment and fertility preferences of married women: analysis of demographic and health survey'2016 in Timor-Leste

Running title: Women's empowerment and fertility preferences: Timor-Leste

Nandeeta Samad[1], Pranta Das[2], Segufta Dilshad[1], Hasan Al Banna[3], Golam Rabbani[4], Temitayo Eniola Sodunke[5], Timothy Craig Hardcastle[6], Ahsanul Haq[7], Khandaker Anika Afroz[8], Rahnuma Ahmad[9] and Mainul Haque[10,*]

[1] Department of Public Health, North South University, Dhaka, Bangladesh
[2] Department of Statistics, University of Dhaka, Dhaka, Bangladesh
[3] Institute of Social Welfare and Research, University of Dhaka, Dhaka, Bangladesh
[4] Health Systems and Population Studies Division, International Centre for Diarrhoeal Disease Research, Bangladesh (icddr,b), Dhaka, Bangladesh
[5] Department of Anatomy, University of Ilorin, Nigeria
[6] Department of Surgery, University of KwaZulu-Natal. South Africa
[7] Gonoshasthaya-RNA Molecular Diagnostic & Research Center, Dhanmondi, Dhaka-1205, Bangladesh
[8] Deputy Manager (Former), Monitoring, Learning, and Evaluation, CEP, BRAC, Bangladesh
[9] Department of Physiology, Medical College for Women and Hospital, Dhaka, Bangladesh
[10] Unit of Pharmacology, Faculty of Medicine and Defence Health, Universiti Pertahanan Nasional Malaysia

* **Correspondence:** Email: runurono@gmail.com, mainul@upnm.edu.my.

Supplementary

Table 1. Odds of fertility preference among the participants who had less than 3 living children.

Variables	No more	Undecided		Have another	
Mother information		OR (95% CI)	p-value	OR (95% CI)	p-value
Age, in years					
25–34	1	1		1	
15–24	1	2.05 (1.23, 3.42)	0.005	2.64 (1.62, 4.31)	<0.001
35–44	1	0.23 (0.17, 0.30)	<0.001	0.08 (0.06, 0.11)	<0.001
45 and above	1	0.11 (0.07, 0.17)	<0.001	0.02 (0.01, 0.03)	<0.001
Education					
No education	1	1		1	
Primary	1	0.67 (0.51, 0.89)	0.005	1.90 (1.26, 2.86)	0.002
Secondary	1	0.69 (0.54, 0.88)	0.002	1.79 (1.27, 2.51)	0.001
Higher	1	0.60 (0.42, 0.86)	0.005	0.33 (0.19, 0.58)	<0.001
Employment status					
Unemployed	1	1		1	
Employed	1	0.99 (0.76, 1.27)	0.921	0.88 (0.68, 1.13)	0.322
Husband information					
Education level					
No education	1	1		1	
Primary	1	1.90 (1.45, 2.48)	<0.001	1.40 (0.96, 2.05)	0.079
Secondary	1	1.93 (1.52, 2.44)	<0.001	2.05 (1.43, 2.92)	<0.001
Higher	1	0.46 (0.33, 0.64)	<0.001	2.64 (1.60, 4.35)	<0.001
Employment status					
Unemployed	1	1		1	
Agricultural work	1	1.05 (0.80, 1.34)	0.788	0.70 (0.47, 1.03)	0.071
Non-agricultural work	1	1.49 (1.19, 1.88)	<0.001	1.43 (1.02, 1.99)	0.037
Household socioeconomic status					
Poorest	1	1		1	
Poor	1	1.03 (0.78, 1.36)	0.832	1.23 (0.79, 1.92)	0.356
Middle	1	1.14 (0.86, 1.51)	0.353	1.21 (0.79, 1.86)	0.385
Richer	1	1.40 (1.05, 1.86)	0.020	1.21 (0.79, 1.84)	0.382
Richest	1	1.23 (0.91, 1.67)	0.168	1.23 (0.79, 1.92)	0.354
Decision					
Do not participate	1	1		1	
Participate	1	0.96 (0.65, 1.40)	0.823	1.00 (0.78, 1.28)	0.989
Contraceptive use					
No	1	1		1	
Yes	1	0.64 (0.54, 0.77)	<0.001	0.54 (0.45, 0.72)	<0.001

*Note: Data was shown as OR with a 95% confidence interval. The multivariate logistic regression model was used to estimate the OR and p-values. The regression model was performed after including all the variables in the model.

Table 2. Odds of fertility preference among the participants who had more than 3 living children.

Variables		No more	Undecided		Have another	
Mother information			OR (95% CI)	p-value	OR (95% CI)	p-value
Age, in years						
	25–34	1	1		1	
	15–24	1	3.50 (0.93, 4.50)	0.058	4.3 (1.27, 5.30)	0.029
	35–45	1	0.52 (0.43, 0.63)	<0.001	0.22 (0.18, 0.28)	<0.001
	45 and above	1	0.30 (0.23, 0.39)	<0.001	0.06 (0.04, 0.10)	<0.001
Education						
	No education	1	1		1	
	Primary	1	0.78 (0.63, 0.97)	0.029	0.80 (0.59, 1.09)	0.161
	Secondary	1	1.08 (0.86, 1.35)	0.505	1.38 (1.02, 4.87)	0.034
	Higher	1	1.63 (1.00, 2.66)	0.051	0.31 (0.17, 0.57)	<0.001
Employment status						
	Unemployed	1	1		1	
	Employed	1	0.76 (0.64, 0.90)	0.001	0.71 (0.57, 0.90)	0.003
Husband information						
	Education level					
	No education	1	1		1	
	Primary	1	0.93 (0.75, 1.19)	0.561	1.08 (0.80, 1.46)	0.615
	Secondary	1	1.02 (0.83, 1.27)	0.831	1.21 (0.89, 1.62)	0.208
	Higher	1	1.14 (0.79, 1.65)	0.491	0.61 (0.38, 0.97)	0.038
Employment status						
	Unemployed	1	1		1	
	Agricultural work	1	1.09 (0.86, 1.38)	0.472	0.92 (0.67, 1.26)	0.609
	Non-agricultural work	1	1.12 (0.89, 1.40)	0.338	1.04 (0.77, 1.40)	0.806
Household socioeconomic status						
	Poorest	1	1		1	
	Poor	1	0.94 (0.73, 1.22)	0.649	1.12 (0.79, 1.57)	0.513
	Middle	1	0.99 (0.76, 1.27)	0.910	0.99 (0.70, 1.39)	0.937
	Richer	1	0.78 (0.59, 1.02)	0.072	0.90 (0.64, 1.28)	0.577
	Richest	1	0.71 (0.52, 0.96)	0.026	0.80 (0.54, 1.20)	0.279
Decision						
	Do not participate	1	1		1	
	Participate	1	1.02 (0.79, 1.32)	0.884	1.13 (0.79, 1.62)	0.498
Contraceptive use						
	No	1	1		1	
	Yes	1	0.51 (0.42, 0.61)	<0.001	0.72 (0.58, 0.90)	0.005

*Note: Data was shown as OR with a 95% confidence interval. The multivariate logistic regression model was used to estimate the OR and p-values. The regression model was performed after including all the variables in the model.

AIMS Press

© 2022 the Author(s), licensee AIMS Press. This is an open access article distributed under the terms of the Creative Commons Attribution License (http://creativecommons.org/licenses/by/4.0)
